# COVID-19-Related Rhino-Orbito-Cerebral Mucormycosis Complicated by the Optic Nerve and Optic Tract Ischemia With Ischemic Neuropathy

**DOI:** 10.7759/cureus.23068

**Published:** 2022-03-11

**Authors:** Stella Onyi, Joon Shin, Chukwuemeka A Umeh, Shyamsunder Sabat, Mehmet S Albayram

**Affiliations:** 1 Radiology, University of Florida College of Medicine, Gainesville, USA; 2 Internal Medicine, Hemet Global Medical Center, Hemet, USA

**Keywords:** ischemic neuropathy, optic tract ischemia, optic nerve ischemia, rhino-orbito-cerebral mucormycosis, covid-19

## Abstract

Mucormycosis is a life-threatening invasive fungal infection usually seen in immunocompromised patients and patients with poorly controlled diabetes mellitus with or without diabetic ketoacidosis. We present a rhino-orbito-cerebral mucormycosis (ROCM) case in a coronavirus disease 2019 (COVID-19) patient complicated by the optic nerve and optic tract ischemia with ischemic neuropathy. Both CT and MRI played an essential role in diagnosing ROCM and the accompanying complications in our patient.

CT showed sinonasal sinusitis and MRI showed the sinusitis and its progression to ROCM. MRI also showed necrosis involving the bilateral orbits, basal ganglia, thalamus, internal capsule, hypothalamus, optic chiasm, optic nerves, olfactory bulbs, and skull base. ROCM associated with optic nerve ischemia is a rare but life-threatening complication of COVID-19, especially in patients with underlying diabetes and/or those treated with corticosteroids. Physicians should be aware of this complication as early diagnosis may improve the chances of survival in such patients.

## Introduction

Mucormycosis is a life-threatening invasive fungal infection caused by fungi of the class mucormycetes [1,2,3]. Mucormycetes are widely distributed in the environment and are opportunistic pathogens that cause severe infection in immunocompromised patients, patients with poorly controlled diabetes mellitus with or without diabetic ketoacidosis, and those with iron overload [3]. Based on clinical presentation and anatomic localization, mucormycosis can be classified into (1) rhinocerebral, (2) pulmonary, (3) cutaneous, (4) gastrointestinal, (5) disseminated, and (6) uncommon/miscellaneous presentations, with rhinocerebral being the commonest type [1,2].

There are few rhino-orbito-cerebral mucormycosis cases in the literature with associated optic nerve ischemia [4-8]. There are even fewer cases of rhino-orbito-cerebral mucormycosis associated with optic nerve ischemia and ischemic neuropathy in the literature [9,10]. With the outbreak of coronavirus disease 2019 (COVID-19), there have been reported cases of acute invasive rhino-orbito-cerebral mucormycosis in the setting of COVID-19 [11-13]. Additionally, multiple instances of mucormycosis in COVID-19 patients with poor glycemic control have been reported, with one multicentric study demonstrating mucormycosis in 1.8% of COVID-19 patients [14]. However, there has been no reported case of optic nerve ischemia with ischemic neuropathy in a COVID-19 patient with mucormycosis to the best of our knowledge.

## Case presentation

A 41-year-old female with underlying liver cirrhosis, type 2 diabetes mellitus, and COVID-19 diagnosed two weeks before hospital admission (symptomatic for five days and treated by a primary care physician with azithromycin and steroids with progressive improvement) and transferred to the emergency department from another hospital for complete vision loss of both eyes. The patient stated that two days before presentation to the hospital, she felt a fleck hit her left eye while barbecuing outside, and she rubbed her eyes without giving it much thought. Later, her left eye started to swell with acute left eye vision loss, and one day before hospital presentation, she subsequently developed right eye vision loss. Upon arrival to the initial hospital, CT showed left retro-orbital and preseptal cellulitis with medial rectus myositis, mild proptosis, left-sided rhinosinusitis, and non-visualization of the left ophthalmic artery for which the patient was started on heparin drip and vancomycin with cefepime. The patient was then transferred to our institution the next day for further workup and evaluation. The patient reported no vision in both eyes and reported pressure behind her eyes, pain associated with extraocular movements, and numbness on the top part of her left forehead. The patient denied any discharge from the eyes or nose.

Vitals on admission were temperature of 102.4 F, a pulse rate of 124 beats per minute, respiratory rate of 25 per minute, blood pressure of 122/60 mmHg, and saturating 100% on room air. Ophthalmologic examination demonstrated the right pupil was 4mm and fixed, left pupil was 5mm and fixed, and elevated intraocular pressure of 26 mmHg in the left eye with normal pressure in the right eye. There was no light perception in both eyes, and the left eye had total ophthalmoplegia, anesthesia within the V1/V2 distribution, pulsatile proptosis, and resistance to retropulsion. The anterior exam was remarkable for lagophthalmos of the left eye, and dilated fundus exam demonstrated central retinal artery occlusion of the left eye. The other physical examination on admission was unremarkable.

Abnormal laboratory results on admission include elevated serum glucose at 214 mg/dl, elevated hemoglobin A1C at 7.3, severe acute respiratory syndrome coronavirus 2 (SARS-CoV-2) antibody was detected, elevated prothrombin time at 23.5 seconds, elevated partial thrombin time at >150, elevated international normalized ratio (INR) at 2.1, decreased albumin at 2.8 g/dl, elevated total bilirubin at 12.7 mg/dl, elevated direct bilirubin at 6.6 mg/dl, elevated alkaline phosphatase at 224 IU/L, elevated aspartate transaminase at 70IU/L, and elevated alkaline transaminase at 52IU/L. Additional abnormal laboratory results include elevated WBC at 12,800/cumm, decreased platelet count at 96,000/cumm, decreased sodium 126 mmol/l, and decreased chloride at 92.

**Figure 1:**
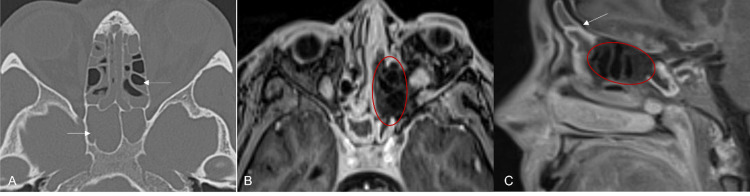
Diffuse sinusitis with necrosis (A) Non-contrast CT bone kernel: diffuse sinus disease centered in the sphenoid and ethmoid sinuses (white arrows); (B) T1 Post-gadolinium MPRAGE: non-enhancing mucosa of left sphenoid sinus (red oval) consistent with necrosis; (C) T1FS post-gadolinium SPACE: partially seen left frontal sinus disease (white arrow) and redemonstration of necrosis of the left ethmoid sinus necrosis (red oval). MPRAGE: magnetization-prepared rapid acquisition gradient echo; T1FS: T1-weighted fat-suppressed; SPACE: sampling perfection with application-optimized contrasts using different flip angle evolution

**Figure 2:**
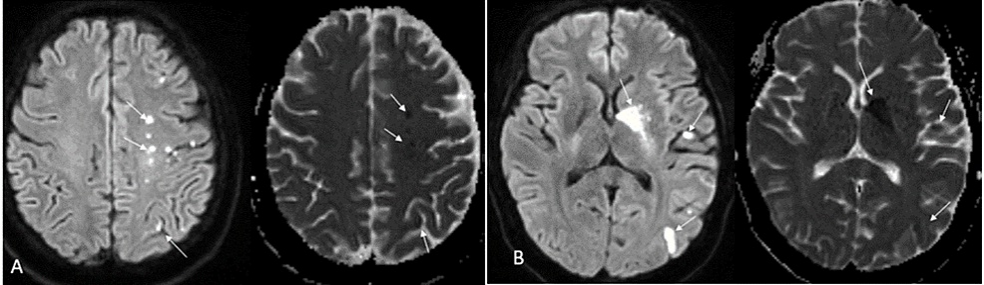
Diffusion-weighted imaging demonstrating left basal ganglia and left hemispheric watershed infarcts (A) Diffusion-weighted imaging demonstrating left-hemispheric watershed infarcts; (B) left basal ganglia and additional watershed areas of infarcts.

**Figure 3:**
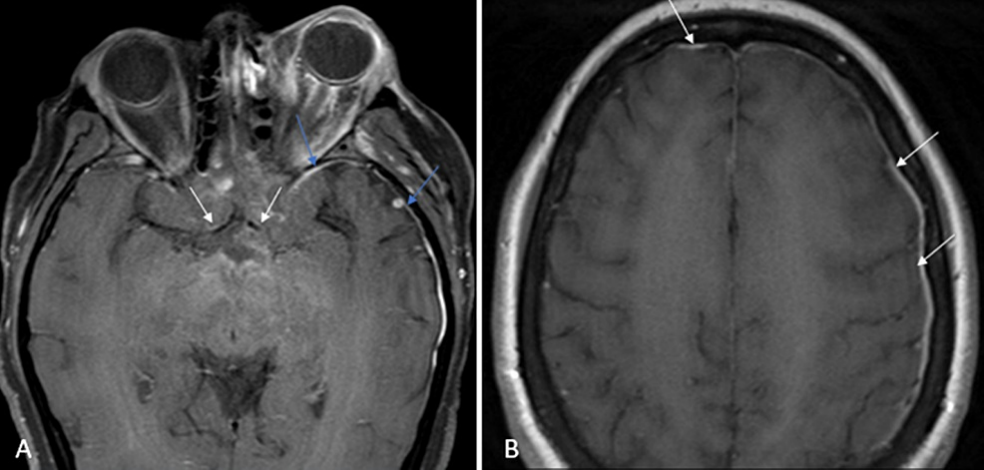
Dural and vascular involvement (A) Axial T1FS TSE post-contrast images demonstrating abnormal perivascular enhancement of the bilateral A1 segments of the anterior cerebral arteries (white arrows) with dural thickening and enhancement (blue arrows); (B) Axial T1 TSE and Axial T1FS TSE post-contrast images demonstrating dural thickening and enhancement. T1FS TSE is T1-weighted fat-suppressed turbo spin echo; T1 TSE: T1 turbo spin echo

Non-contrast CT was obtained demonstrating pansinusitis (Figure 1). Additionally, an MRI of the brain was obtained to further evaluate the abnormal findings on CT and demonstrated multiple ischemic changes in the left hemispheric watershed territories and left basal ganglia. There was also abnormal meningeal thickening and enhancement with abnormal luminal narrowing and wall enhancement of the internal carotid artery, middle cerebral artery, and anterior cerebral artery suspicious for infectious/inflammatory arteriopathy. There was also abnormal parenchymal contrast enhancement involving the hypothalamic region and base of the basal ganglia with periventricular involvement suggestive of an extensive infectious and inflammatory process involving multi compartments with etiology most likely related directly or/and indirectly to COVID-19 (Figure 2, Figure 3).

**Figure 4:**
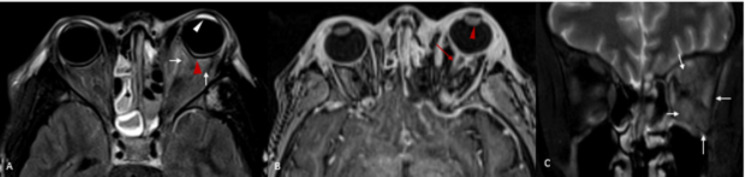
Intra-orbital and optic pathway involvement (A) T2 FLAIR post-gadolinium: diffuse inflammation of left post-septal compartment (white arrows) with left orbital proptosis and tenting of posterior globe (red arrowhead), additional abnormal signal in the left anterior chamber (white arrowhead); B) T1 post-gadolinium MPRAGE: enlarged left lens (red arrowhead) and enhancement and prominence of the left optic nerve sheath (red arrow); C) T2WI coronal: diffuse inflammation of left extra-ocular muscles (white arrows). FLAIR: fluid-attenuated inversion recovery; T2WI: T2 weighted image; MPRAGE: magnetization-prepared rapid acquisition gradient echo

**Figure 5:**
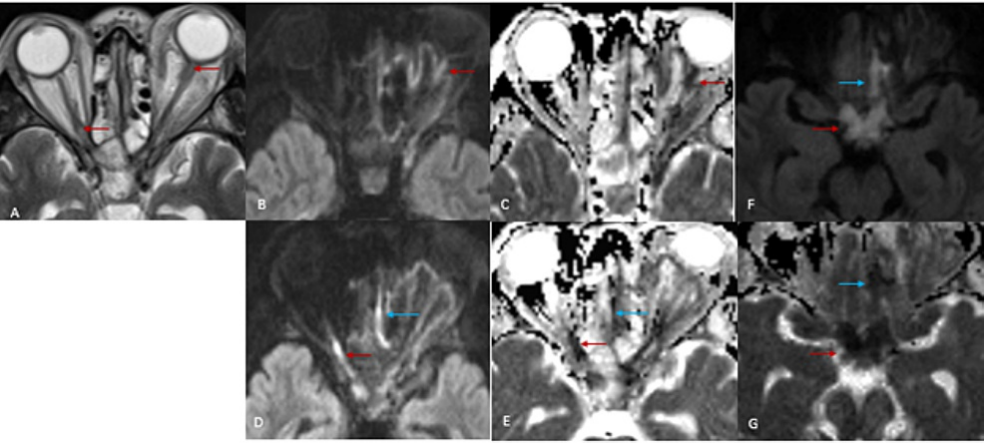
Intra-orbital and optic pathway involvement continued (A)-(E): Bilateral optic nerve involvement: (A) T2WI for anatomy; red arrows correspond to diffusion restriction on (B) to (E);  (B)-(E) DWI and ADC: diffusion restriction of left and right optic nerves (red arrows). Also noted diffusion restriction in the left olfactory tract (blue arrows); (F)-(G): DWI and ADC: diffusion restriction in the optic chiasm (red arrows) and left gyrus rectus along the olfactory tract (blue arrows) DWI: diffusion weighted imaging; ADC: apparent diffusion coefficient; T2WI: T2 weighted image

MR orbit was obtained demonstrating abnormal bilateral thickening, contrast enhancement, and T2 signal increase of the globe layers, anterior chamber, lens, retro-orbital fat, and extraocular muscles. In addition, there was abnormal bilateral extensive thickening of the optic nerve, optic sheath, optic chiasm, and optic tracts with associated abnormal contrast enhancement and diffusion restriction, left greater than right. There was also abnormal contrast enhancement and signal increase of the left olfactory bulb and gyrus rectus. Pan sinusitis was also noted (Figure 4, Figure 5).

**Figure 6:**
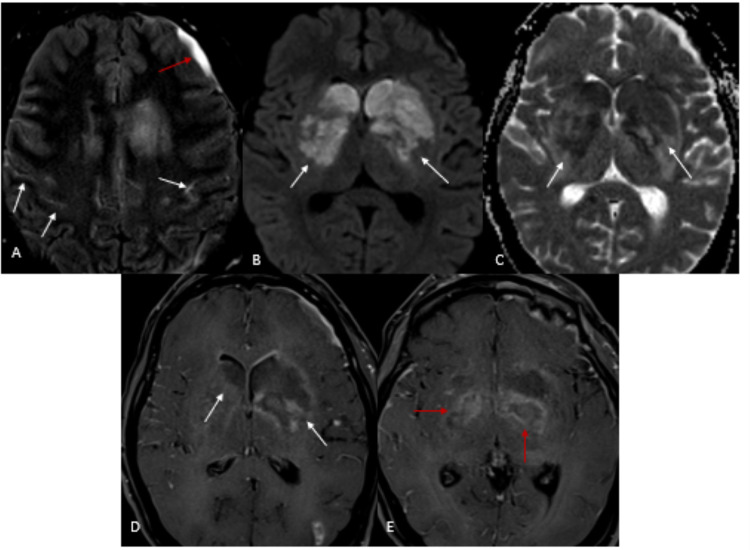
Intracranial extension of the mucormycosis (A) T2 FLAIR post-gadolinium: leptomeningeal enhancement and FLAIR-nonsuppression (white arrows) and subdural collections (red arrow); (B)-(C) DWI and ADC: diffusion restriction in the deep nuclei bilaterally (white arrows); (D)-(E) T1 post-gadolinium MPRAGE: large necrotic foci in the left greater than right basal ganglia (white arrows) and the hypothalamus (red arrows). FLAIR: fluid-attenuated inversion recovery; MPRAGE: magnetization-prepared rapid acquisition gradient echo; DWI: diffusion weighted imaging; ADC: apparent diffusion coefficient

**Figure 7:**
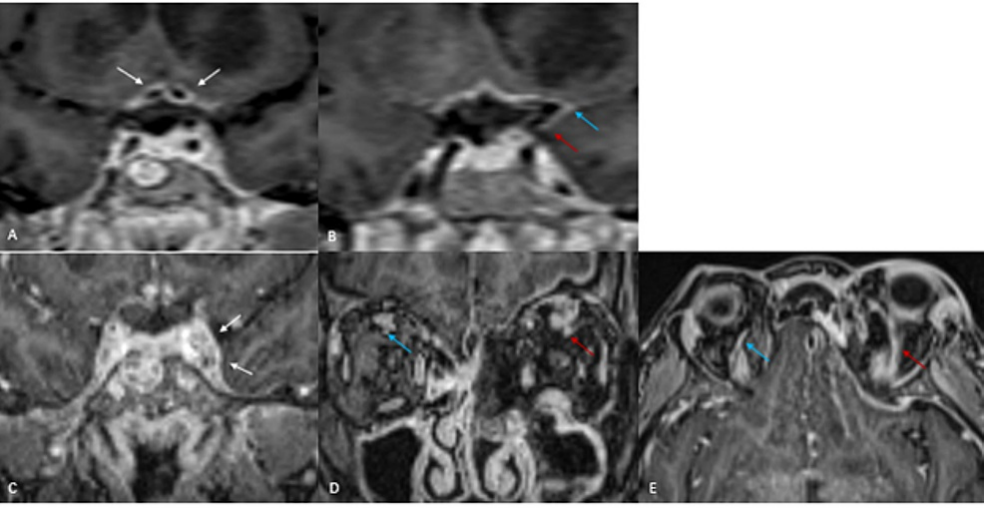
Intracranial thrombophlebitis and vasculitis (A)-(B) T1 post-gadolinium MPRAGE coronal: Periarterial enhancement of bilateral A1 (white arrows), left supraclinoid internal carotid artery (red arrow), and the left M1 (blue arrow); (C)-(E) T1 post-gadolinium MPRAGE: Convex bulging of the left cavernous sinus (white arrows) and prominent left superior ophthalmic vein (red arrows) raising concern for thrombophlebitis. Normal right superior ophthalmic vein (blue arrows) for comparison. MPRAGE: magnetization-prepared rapid acquisition gradient echo

The patient was admitted to the intensive care unit and started on intravenous cefepime, vancomycin, metronidazole, and heparin drip. Both dexamethasone and remdesivir were not given for COVID-19 as the patient did not meet the criteria. The neurosurgeon performed catheter angiography and found cavernous sinus thrombosis and the left ophthalmic artery occlusion. The patient’s mental status progressively declined while on admission and on day seven of admission, the Glasgow coma scale declined from 11 to 7, and the patient was emergently intubated to protect her airway. Magnetic resonance angiography (MRA) and magnetic resonance venography (MRV) were obtained on day 10 of admission and demonstrated progression of extensive multifocal inflammatory and infectious changes with interval development of necrosis involving the bilateral orbits, basal ganglia, thalamus, internal capsule, hypothalamus, optic chiasm, optic nerves, olfactory bulbs, and skull base. In addition, there was periarterial enhancement and thrombophlebitis. There was also interval worsening of the left paranasal sinusitis with extensive necrotic inflammatory changes involving the left ethmoidal, sphenoidal, and maxillary sinuses and sinus walls with extension into the left superior orbital fissure, left optic foramen, and inferior orbital fissure suspicious for infectious disease such as invasive fungal sinusitis including mucormycosis (Figure 6, Figure 7).

Nasopharyngoscopy demonstrated gray necrotic nasal tissue; frozen biopsy revealed broad hyphae. The patient was started on amphotericin and voriconazole empirically on day 11 of admission. Tissue culture later identified the organism as *Rhizopus*, and a decision was made to switch from voriconazole to posaconazole, the first-line antifungal for mucormycosis. However, given planned palliative extubation due to the patient’s poor prognosis, posaconazole was held off as it was unlikely to change this patient's course given her advanced invasive disease. An Ear, Nose, and Thorat (ENT) surgeon was consulted and he recommended against surgical debridement because it will not improve the patient’s overall poor prognosis. The patient was compassionately extubated and placed on comfort care based on her family and medical team's decision that further treatment was futile and the patient expired on day 15 of admission.

## Discussion

Coronaviruses are single-stranded, positive, enveloped RNA viruses of which SARS-CoV-2 is part [15]. SARS-CoV-2 infects the human body through membranes that exhibit angiotensin-converting enzyme 2 (ACE2) receptors [16,17]. There have been several reported cases of mucormycosis in COVID-19 patients, and risk factors for mucormycosis in COVID-19 patients include diabetes or steroid-induced hyperglycemia, high iron and ferritin levels, steroid-induced immunosuppression, and prolonged hospitalization [3,18,19]. Infection usually occurs through inhalation of the spores, which invade the nasal mucosa and sinuses, causing rhinosinusitis [11].

Mucormycosis usually presents with engorgement of the turbinates, ischemia, and thrombosis of the turbinates leading to necrosis [20]. Affected individuals at the early stages can present with fever, acute sinusitis, nasal congestion, purulent nasal discharge, and headache [14]. If not diagnosed and treated early, mucormycosis easily spreads to the brain by either direct extension through the cribriform plate, infratemporal fossa, inferior orbital fissure, and walls of the sinuses or the invasion of the arteries and veins with resultant vascular thrombosis and infarction [11,14,20,21]. The symptoms of intracranial infection include diplopia, ophthalmoplegia, acute vision loss, cranial nerve deficits, focal neurological deficits, and altered sensorium [11]. Sudden onset vision loss in rhino-orbito-cerebral mucormycosis has been associated with central retinal artery occlusion, ophthalmic artery necrosis, optic nerve infarction, or direct optic nerve infection [7]. Survival with intracerebral involvement is noted to be rare [22].

MRI is more sensitive than CT for detecting rhino-orbito-cerebral mucormycosis [12]. On MRI, the presence of nasal, sinus, and orbital tissue necrosis suggests mucormycosis [7]; however, microbiological and histologic evidence of mucormycetes is required to confirm the diagnosis [12,13]. Our patient presented with COVID-19 and acute vision loss and was found to have occlusion of the left ophthalmic artery, abnormal luminal narrowing, and wall enhancement of the intracranial arteries with associated optic nerve restricted diffusion and multiple areas of brain parenchymal restricted diffusion on the MRI, suggesting ischemia/infarct. Our patient subsequently developed rhino-orbito-cerebral mucormycosis demonstrated by necrosis of the nasal turbinates, paranasal sinuses, orbits, and brain parenchyma.

Treatment of mucormycosis is multifactorial and involves early diagnosis, treatment of underlying or contributory causes such as metabolic acidosis, treatment with antifungals, and surgical debridement [23]. The first step to successfully managing mucormycosis is early detection of the disease. Early detection in COVID-19 patients requires a high index of suspicion, especially in patients with diabetes who received corticosteroids [14]. Acute sinusitis, purulent nasal discharge, vision loss, or cranial nerve deficits in COVID-19 patients should be red flags for investigating mucormycosis, especially in diabetic patients receiving corticosteroids. Unfortunately, many cases of mucormycosis in COVID-19 patients are diagnosed late because eschar formation, the hallmark of mucormycosis, is often a late sign [14]. Thus, a low threshold for investigation and early empiric treatment based on strong clinical suspicion will be essential to avoid the complications and high mortality associated with mucormycosis in COVID-19 patients.

First-line antifungal monotherapy options include amphotericin B (liposomal) or posaconazole oral suspension [14]. In addition, posaconazole can be combined with liposomal amphotericin B in refractory cases. Our patient received antifungals which were started on day 11 of admission; however, the patient family and the medical team decided to discontinue treatment and place the patient on comfort care as treatment was deemed futile due to extensive brain parenchymal, orbital, sinus, and nasal inflammation and necrosis. Infection is usually rapidly progressive and fatal unless early aggressive treatment with antifungal agents and surgical excision is started. If left untreated, mucormycosis could be fatal in more than 50% of cases [24].

## Conclusions

In conclusion, we present a case of rhino-orbito-cerebral mucormycosis with associated optic nerve ischemia, a rare but life-threatening complication of COVID-19, especially in patients with underlying diabetes and/or those treated with corticosteroids. MRI played an important role in diagnosing this condition and the accompanying complications in our patient. Physicians should be aware of this complication as early diagnosis may improve the chances of survival in such patients.
